# Does the pain-protective GTP cyclohydrolase haplotype significantly alter the pattern or severity of pain in humans with chronic pancreatitis?

**DOI:** 10.1186/1744-8069-4-58

**Published:** 2008-11-17

**Authors:** Mark Lazarev, Janette Lamb, M Michael Barmada, Feng Dai, Michelle A Anderson, Mitchell B Max, David C Whitcomb, the NAPS2 Consortium

**Affiliations:** 1 Departments of Medicine, University of Pittsburgh, Pittsburgh, PA 15213, USA; 2 Human Genetics, University of Pittsburgh, Pittsburgh, PA 15213, USA; 3 Anesthesiology, University of Pittsburgh, Pittsburgh, PA 15213, USA; 4 Cell Biology and Physiology, University of Pittsburgh, Pittsburgh, PA 15213, USA; 5 Department of Medicine, University of Michigan, Ann Arbor Michigan, USA; 6 UPMC Presbyterian, M2 C-Wing, 200 Lothrop Street, Pittsburgh, PA 15213, USA

## Abstract

**Background:**

Pain is often a dominant clinical feature of chronic pancreatitis but the frequency and severity is highly variable between subjects. We hypothesized that genetic polymorphisms contribute to variations in clinical pain patterns. Since genetic variations in the GTP cyclohydrolase (GCH1) gene have been reported to protect some patients from pain, we investigated the effect of the "pain protective haplotype" in well characterized patients with chronic pancreatitis (CP) or recurrent acute pancreatitis (RAP) from the North American Pancreatitis Study 2 (NAPS2).

**Results:**

Subjects in the NAPS2 study were asked to rank their pain in one of 5 categories reflecting different levels of pain frequency and severity. All subjects were genotyped at rs8007267 and rs3783641 to determine the frequency of the GCH1 pain-protective haplotype. In Caucasian subjects the frequency of the pain-protective GCH1 haplotype was no different in the control group (n = 236), CP patients (n = 265), RAP patients (N = 131), or in CP patients subclassified by pain category compared to previously reported haplotype frequencies in the general Caucasian population.

**Conclusion:**

The GCH1 pain-protective haplotype does not have a significant effect on pain patterns or severity in RAP or CP. These results are important for helping to define the regulators of visceral pain, and to distinguish different mechanisms of pain.

## Introduction

Chronic pancreatitis (CP) is a chronic inflammatory disorder of the pancreas characterized by progressive destruction of the parenchyma, loss of exocrine and endocrine function, and in many cases, severe abdominal pain [1,2]. The problem of pain in CP is complex and has been difficult to study because of the multiple contributing etiologies, the variable frequency and severity of pain, and other challenges that are common in the study of visceral pain [3,4]. The commonly recognized etiologies and treatments have been reviewed elsewhere [3]. However, the possibility that a component of the variance in pancreatic pain severity or chronicity is genetically determined has not been explored.

Based on clinical experience in Zurich Switzerland, Ammon et al describe two distinct patterns of pain in patients with alcoholic CP (>90% males) as A Type (episodic pain with pain-free intervals) and B Type (continuous pain with exacerbations) [5]. B-type pain was considered the most severe, requiring significantly more hospitalizations and surgical interventions for attempted pain relief. These findings are consistent with pain in other disorders where pain severity correlated with interference in daily functioning [6].

Tegeder et al. [7] reported that specific genetic variations in the GTP cyclohydrolase (GCH1) gene were associated with reduced severity of persistent leg pain in Caucasian patients with chronic radicular disease who underwent diskectomy, as well as with lower ratings of experimental pain stimuli in normal young adults. GCH1 is the rate limiting enzyme in the production of 6(R)-L-*erythro*-5,6,7,8-tetrahydrobiopterin (BH4). BH4 is a key cofactor in the synthesis of several pain neuromodulators including catecholamines, serotonin and nitric oxide and is important in the metabolism of phenylalanine [8]. BH4 appears to increase pain sensitivity by upregulation of nitric oxide [7]. Therefore, changes in GCH1 activity would alter BH4 levels and could thereby modulate pain signaling. Tegeder et al. discovered an uncommon haplotype in the GCH1 gene (termed the pain-protective haplotype) found in about 15% of Caucasians' chromosomes containing 5 single nucleotide polymorphisms (SNPs) significantly associated with low pain, including rs8007267G>A and rs3783641A>T [7,9]. Whether the same "pain-protective haplotype" seen in this neuropathic and experimental pain study alters pain severity or frequency in visceral pain syndromes, including CP, is unknown.

We tested the hypothesis that the "pain-protective haplotype" is protective for CP pain severity and chronicity. Thus, patients with this haplotype would be predicted to be more likely to have mild and/or intermittent patterns of pain. The patient population included adult subjects with recurrent acute pancreatitis (RAP), CP and controls from the North American Pancreatitis Study 2 (NAPS2).

## Methods

### Clinical cohort

NAPS2 is the largest prospective molecular epidemiology and genetics study of RAP and CP in the United States with recruitment of nearly 1700 subjects from 20 expert centers [10]. All patients were phenotyped by physicians with an expertise in pancreatic disease. Additionally, custom questionnaires that included demographic, environmental, comorbid, phenotypic, quality of life and pain questions were filled out by both patients and physicians [10].

The final NAPS2 cohort included 460 subjects with RAP, 540 patients with CP, and 695 healthy controls. Of the 1000 total patients, 540 had completed a pain questionnaire and provided a DNA sample. Eighty-one patients were excluded because of ambiguous TaqMan^® ^SNP genotyping (see methods) results, while an additional 63 patients were excluded because they listed their race as 'African American' or 'Other'. Non-Caucasian populations are known to have a higher frequency of the uncommon allele for both SNPs: 50% for SNP1 - rs8007267G>A and 23.8% for SNP2 - rs3783641A>T in our sample, and have a markedly different pattern of haplotypes, compared to Caucasians. The final cohort used for analysis included 396 Caucasian patients (265 CP, 131 RAP).

459 healthy controls were identified who were either spouses of the patients or unrelated individuals without a history of pancreatic disease or diabetes. Two hundred-sixty controls were randomly chosen, 16 were excluded for ambiguous genotyping data, and 8 non-Caucasians were excluded. The final subgroup used for analysis included 236 Caucasian controls.

### Pain questionnaire

Constant or intermittent episodes of severe pain tend to be most disruptive to CP patients' lives. The pain questionnaire for the NAPS2 study was expanded to include severity by describing 5 pain conditions based on both pattern (chronicity) and severity (Table 1) [10]. Subjects were asked to provide the one best answer that describes the pancreatitis-associated symptoms. The scale allowed subjects to be categorized by specific pain response, and by binary analysis for severity (A+B+C {mild} vs. D+E {severe}) or chronicity (A+C {intermittent} vs. B+D+E {constant}). For example, a patient with Group 'A' pain has significantly less pain than a patient with Group 'E' pain – both in terms of severity and chronicity. Both the severity and chronicity measures correlate with quality of life as measured using the Short Form 12 (SF12), (manuscript in preparation). Fifty-nine subjects (14.9%) were found to have A pain, 28 (7.1%) B pain, 125 (31.6%) C pain, 142 (35.9%) D pain, and 41 (10.4%) E pain.

**Table 1:** 

Chronic pancreatitis pain questions from the NAPS2 study [10].
□ A) I have episodes of mild to moderate pain, usually controlled by the medicines noted above.
□ B) I have constant mild to moderate pain, usually controlled by medicines noted above.
□ C) I am usually free of abdominal pain, but I have episodes of severe pain.
□ D) I have constant mild pain that is controlled (as above), plus episodes of severe pain.
□ E) I have constant severe pain that does not change.

Patients in the NAPS2 study were given 5 choices from which to answer the following question. "Several patterns of pain have been described in chronic pancreatitis. In this question, please identify the type of pain that best fits your condition"

### Genotyping

Five SNPs within the Chr14q22.1-q22.2 were used to define the primary *GCH1 *"pain-protective haplotype" by Tegeder et al (see their Figure 6a) [7]. However, the haplotype can be defined by 2 SNPs [9]: SNP1 – dbSNP rs8007267 (c.-9610G>A) and SNP2 – dbSNP rs3783641 (c.343 + 8900A>T) which are located in the promoter region and intron 1, respectively [9]. These two SNPs were therefore used to screen for the "pain-protective haplotype" in patients with CP.

DNA was extracted and quantified as described by Whitcomb [10]. Genotypes were determined using TaqMan^® ^SNP genotyping assays (Applied Biosystems, Foster City, California, USA). The assays are the same as those reported in Tegeder et al. Supplementary Table 3 [7].

Thirty-four subjects (28 patients, 6 controls) with unambiguous TaqMan^® ^SNP genotyping were sequenced for SNP 1 and 2 using Big Dye chemistry (Applied Biosystems, Foster City, California, USA) at a 1:4 dilution, but otherwise according to the manufacturer's directions. There was 100% concordance between the sequencing results and those determined by Taqman genotyping for both common and uncommon alleles. An additional 4 patients were sequenced to resolve ambiguous results obtained from Taqman genotyping. Resolution of ambiguous genotyping results was only performed for Group A, B, and E patients, for whom there were far fewer subjects available compared to Group C and D patients. The primary explanation for ambiguous genotyping results is attributed to poor DNA quality.

### Statistical Analysis

Population haplotype frequencies were estimated by the expectation-maximization (EM) algorithm and Hidden Markov Model (HMM) techniques as implemented in fastPHASE [11]. Comparison of the frequencies of fastPHASE-derived haplotype/diplotype configurations in the case and control groups was examined using Fisher's Exact Test running under R software . Differences were considered to be statistically significant at P < 0.05.

## Results

**Figure 1:**
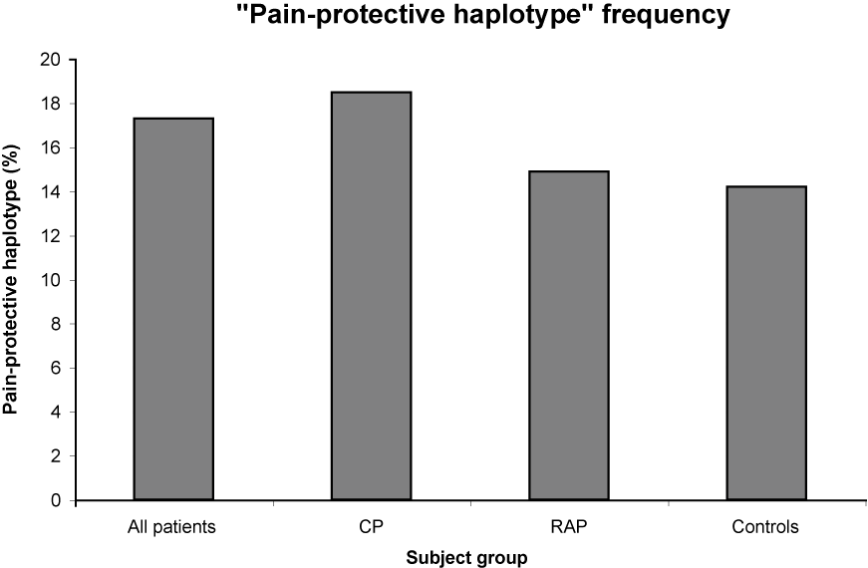
**Pain-protective haplotype frequency among subject groups**. The frequency of the "pain protective haplotype", given as a percent of the total haplotype count. "All patients" includes both CP and RAP. There was no significant difference between groups. "Pain protective haplotype" count: All pts = 137, CP = 98, RAP = 39, Controls = 67.

**Figure 2:**
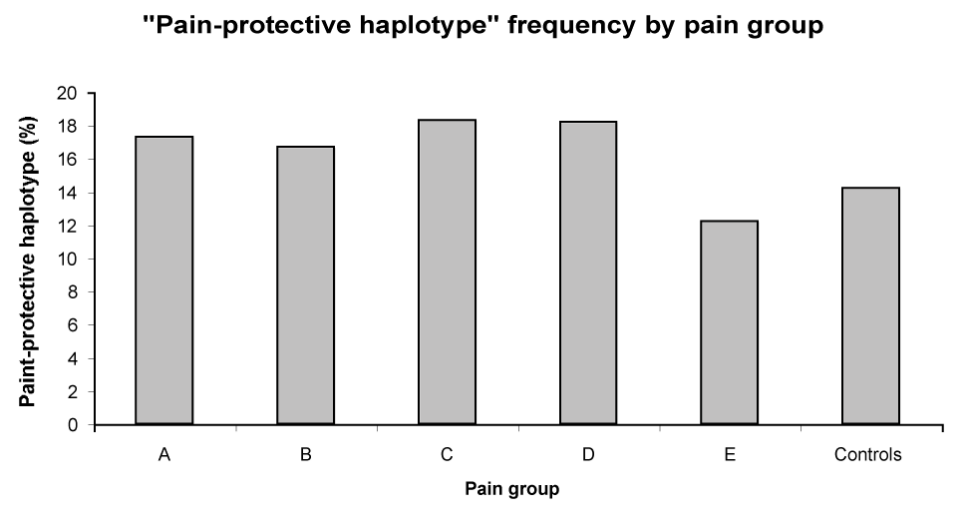
**Pain-protective haplotype frequencies among pain groups**. The frequency of the "pain protective haplotype" as percentage of total counts in the CP and RAP groups subdivided by the subject's response to the pain question. There was no significant difference between groups. "Pain protective haplotype" count: A = 20, B = 9, C = 46, D = 52, E = 10.

**Figure 3:**
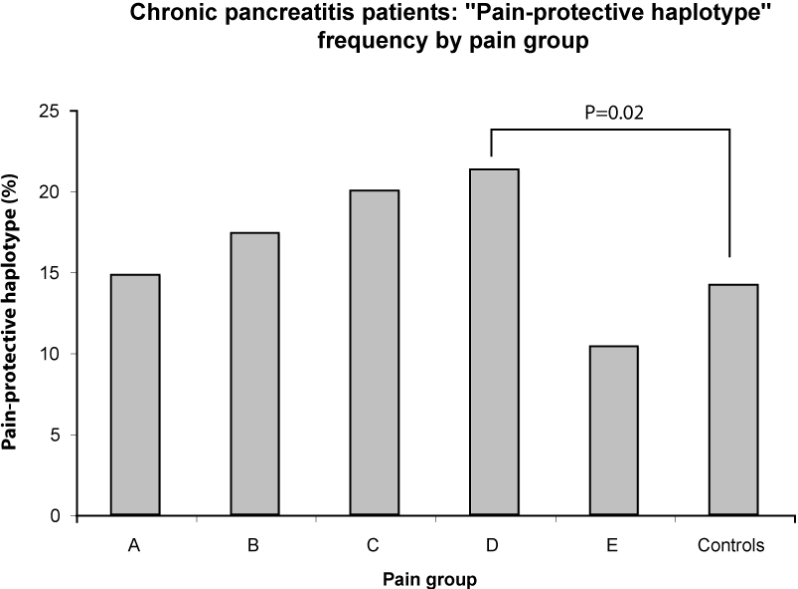
**Pain-protective haplotype frequency in CP by pain type**. The frequency of the "pain protective haplotype" as percentage of total counts in the CP group subdivided by the subject's response to the pain question. * P = 0.02 comparing group D compared to controls. This was in the opposite direction that was hypothesized, and the value became non-significant after Bonferroni corrected for multiple testing. No other significant differences were detected. "Pain protective haplotype" counts: A = 13, B = 8, C = 32, D = 40, E = 5, Controls = 67.

The demographic results for the subjects are presented in Table 2. Using fastPHASE, the common haplotype (GA), the uncommon haplotype (AT – also known as the "pain-protective haplotype"), and other less common haplotypes were determined. Fisher's exact test for the GA and AT haplotypes in RAP and CP versus controls was performed and the p-values recorded. Table 3 shows the haplotype frequencies for all patients, CP patients, RAP patients, healthy controls, as well as the corresponding p-value. Table 4 shows a subdivision of all patients, CP patients, and RAP patients into different pain groups. The only significant finding is the CP patients with group D pain pattern, who were more likely to possess the uncommon AT haplotype in comparison to controls p = 0.02 (OR = 1.69; CI 1.06–2.68)) which became non-significant after Bonferroni corrected for multiple testing. Allelic frequencies of the AT haplotype are graphically displayed for all groups (Figure 1), among different pain groups (Figure 2), and for chronic pancreatitis patients (Figure 3).

**Table 2:** 

	Patients	Controls
Total	396	236
Males	180 (45.5%)	98 (41.5%)
Females	216 (54.5%)	138 (58.5%)
Age	47.2 +/- 16.1	56.9 +/- 14.8
Chronic pancreatitis	265 (66.9%)	0
Recurrent acute pancreatitis	131 (33.1%)	0
Alcohol associated etiology	134 (33.8%)	0
Other etiologies	262 (66.2%)	0
Smoking history	252 (63.6%)	119 (50.4%)

Demographics of subjects in the patient and control groups.

**Table 3:** 

	All pts	CP	RAP	Controls
GA	629	415	214	398
haplotype	(79.4)	(78.3)	(81.7)	(84.3)
				
AT	137	98	39	67
haplotype	(17.3)	(18.5)	(14.9)	(14.2)
				
Other	26	17	9	7
haplotype	(3.3)	(3.2)	(3.4)	(1.5)
				
Fisher	0.11	0.06	0.74	---
P-value				

Haplotype frequency in all subjects formed by rs8007267 and rs3783641. Haplotype count with percentage in parentheses. Fisher p-value takes into account the GA and AT haplotypes only

**Table 4:** 

	All A	All B	All C	All D	All E	CP A	CP B	CP C	CP D	CP E	RAP A	RAP B	RAP C	RAP D	RAP E
GA	95 (80.5)	44 (81.5)	201 (79.8)	218 (76.2)	71 (86.6)	72 (81.8)	37 (80.4)	124 (77.8)	140 (74.5)	42 (87.5)	23 (76.7)	7 (87.5)	77 (83.7)	78 (80.0)	29 (85.3)
AT	20 (17.0)	9 (16.7)	46 (18.3)	52 (18.2)	10 (12.2)	13 (14.8)	8 (17.4)	32 (20)	40 (21.3)	5 (10.4)	7 (23.3)	1 (12.5)	14 (15.2)	12 (12.2)	5 (14.7)
Other	3 (2.5)	1 (1.9)	5 (2.0)	16 (5.6)	1 (1.2)	3 (3.4)	1 (2.2)	4 (2.5)	8 (4.3)	1 (2.1)	0 (0)	0 (0)	1 (1.1)	8 (8.2)	0 (0)
Fisher P-value	0.47	0.68	0.16	0.10	0.73	0.87	0.51	0.08	0.02	0.66	0.19	1.0	0.87	0.87	1.0

Haplotype counts among the subject groups subdivided by the subject's response to the pain question (percentage of total haplotype count in parentheses). Haplotypes are formed by rs8007267 and rs3783641. AT was reported to be the "pain-protective haplotype" [7]. "All" is CP and RAP combined. Fisher's Exact Test compares each category with the haplotype frequencies in the overall control group.

Grouping all patients into intermittent pain (A + C) or constant pain (B + D + E) categories, haplotype frequencies were similar to the control population (p = 0.15 and 0.23, respectively). Similarly, there was no difference compared to controls for mild pain (A + B + C) or severe pain (D + E) – (p = 0.14 and 0.21, respectively). Comparison of intermittent vs. constant pain and mild vs. severe pain were also not significant – p = 0.85 and 0.92, respectively. Separate comparisons of intermittent/continuous and mild/severe pain for patients with CP and RAP also did not reach significance (data not shown).

Eleven of 396 (2.7%) subjects with CP or RAP were identified as being homozygous for "pain-protective haplotype" versus 5 of 236 (2.1%) of controls (p = 0.80). There was no statistically significant variation in the frequency of patients with a homozygous "pain-protective haplotype" among different pain groups (A to E).

## Discussion

This is the first study to evaluate the *GCH1 *"pain protective" haplotype in a visceral pain syndrome. Unlike neuropathic pain which was persistent in the post-operative state, there was no association with the "pain protective" *GCH1 *haplotype and the individual or combined patient group (CP and RAP), or individual pain patterns or pain severity levels in our study. While these findings do not diminish the importance of the previous findings in post-surgical sciatica following diskectomy, it does suggest that the mechanisms of pancreatitis-associated visceral pain may either be different than in persistent post-surgical sciatica, or more heterogeneous.

It is unlikely that the difference between the findings of Tegeder and the present study are due to differences in base populations. Indeed, the frequency of the "pain-protective haplotype" was 17.3% in our patient sample and 15.4% for the patients in the Tegeder study. Similarly, the control frequencies were also similar, being 14.2% vs. 15.8%, respectively.

The strength of the current study includes the relatively large sample size and the ability to distinguish various pain patterns. The limitations include poor understanding of the etiology of the variation in pancreatic pain patterns and severity and limitations in accurate phenotyping, which is based on the response of patients to a questionnaire with only 5 categories to describe pain, administered once during their illness. In contrast, the sciatica study reported in Tegeder et al. [7] assessed pain at three distinct time points, using aggregates scores from four 7-category scales. The pain measurement literature suggests that increases in the number of scale categories above five [12,13] and averaging ratings at 2–7 time points [14] reduces the error in estimating the magnitude of chronic pain, or its responses to drug treatment.

Pain is a complex perception that includes both physiologic and pathologic components, and involves multiple pathways and genes. The discovery that *GCH1 *polymorphisms [7], catechol-O-methyltransferase (COMT) polymorphisms [15,16], and possibly other "pain gene" polymorphisms [15] alter perception and tolerance to pain in humans represent important breakthroughs in understanding the observed clinical differences in reported symptoms between patients with similar pathologies. As noted above, the present study is the first to evaluate the *GCH1 *"pain-protective haplotype" in visceral pain, but our findings do not rule out the possibility that GCHI-dependent pathways play an important role in visceral pain involving other organs, or in small subsets of pancreatitis-associated pain.

The initial published report of *GCH1 *genotypes influencing clinical pain suggested that pain pathways sensitive to changes in GCH1 expression involve neuropathic pain [7], which may be uncommon in RAP or CP. GCH1 expression was significantly increased in a rat model of peripheral neuropathic pain, and blocking GCH1 reduced the pain response [7]. Additionally, the pain-protective *GCH1 *haplotype correlated with decreased persistent leg pain scores in Caucasian patients who underwent diskectomy, with homozygous genotypes correlating with the lowest scores and heterozygote genotypes correlating with intermediate scores [7]. They were also able to replicate this finding in healthy controls who were exposed to mechanical pain. Of note, although the *GCH1 *"pain-protective haplotype" is associated with reduced neuropathic pain, the functional SNP(s) have not been defined. 

The above findings have been replicated in other recent studies. In a study of 10 healthy persons homozygous for the AT haplotype, the investigators found increased mechanical pain thresholds if participants were sensitized with local skin inflammation [17]. In CM Campbell et al. (under review), pain ratings of 39 healthy subjects administered topical capsaicin were lower in those with polymorphisms in the GCH1 gene. Notably, there was no association with SNP 1 (rs8007267) [18].

By contrast, Kim et al. [19] analyzed 38 SNPs in the GCH1 gene with a heterozygosity > 0.2 among 221 subjects who rated pain severity after surgical removal of an impacted third molar. No association was seen between genetic variations in *GCH1 *and pain sensitivity. Different findings among these studies may in part be attributed to variations in experimental stimuli [20].

Multiple etiologies, and therefore multiple pathways likely contribute to pancreatic pain [4]. In some cases, the pattern of pain may provide clues to physiological pain mechanisms. Intermittent pain, for example, is more often associated with blockage of the pancreatic duct, acute pancreatitis, a pseudocyst or other structural lesions [5]. On the other hand, constant pain is usually associated with chronic inflammation or an inflammatory mass [5]. Of note, subjects with severe CP pain and long standing inflammation who subsequently underwent surgery were found to have an increase in the number of nerves, with larger cross-sectional areas than seen in the normal pancreas [21,22]. The perineural nerve sheath may also become damaged by infiltrating lymphocytes, causing a pancreatitis-associated neuritis and neuropathy [23,24]. Additionally, enhanced expression of several neuronal growth factors have been found to be correlated to the severity of pain in CP – including growth associated protein 43, brain derived neurotrophic factor, tyrosine kinase receptor A (the high affinity receptor for nerve growth factor), and artemin [24-28]. These lines of evidence suggest that subjects with CP have inflammation associated pain rather than neuropathic pain.

In summary, the factors responsible for the marked differences in pain perception in RAP and CP with similar pathologies remain unknown. The current study is important, however, in that it helps to define pain pathways and differences in the mechanisms of pain in humans. Taken together, statistical association of a candidate pain gene with a specific pain type will help define the pathways activated in the context of various disorders and injuries, and may help guide future treatments.

## Abbreviations

BH4: 6(R)-L-*erythro*-5,6,7,8-tetrahydrobiopterin; CP: Chronic pancreatitis; COMT: catechol-O-methyltransferase; GCH1: GTP cyclohydrolase 1; NAPS2: North American Pancreatitis Study 2; RAP: recurrent acute pancreatitis; SNP: single nucleotide polymorphism.

## Competing interests

MM is one of the holders of a patent on the use of GCH1 polymorphisms in diagnostic tests.

## Authors' contributions

ML assisted in the design of the study, development of the assays, carried out the laboratory experiments, completed the first draft of the manuscript and participated in all stages of editing. JL supervised the development of the assays, completed the initial data analysis and assisted in writing the methods and results sections of the manuscript. MMB and FD were responsible for statistical analysis and manuscript review. MA and MM assisted in review of the data and wrote parts of the discussion and reviewed multiple drafts of the manuscript. DCW designed the and directed the NAPS2 program that ascertained and phenotyped the subjects, designed the present project with ML and JL, reviewed the data and analysis, and assisted in writing all versions of the manuscript. Physicians who contributed to the patient cohort are listed as members of the NAPS2 consortium in Whitcomb et al [10].

## Authors' information

ML completed this project in partial fulfillment of a clinical gastroenterology fellowship at the University of Pittsburgh and is currently the Present-Levison fellow, Division of Gastroenterology, Mount Sinai School of Medicine, New York, New York.
